# Hyperactive PDGFRβ signaling induces cataractogenesis via TGFβ and STAT5-IGF1

**DOI:** 10.1016/j.ydbio.2025.09.009

**Published:** 2025-09-13

**Authors:** Jesse J. Reardon, Yixuan Ma, Nathaniel S. Grabinski, Yalini Ramamoorthy, Rebecca L. Packard, Johnathon G. Schiebel, Heather L. Chandler, Gina M. Sizemore

**Affiliations:** a The Comprehensive Cancer Center, The Ohio State University, Columbus, OH, USA; b Department of Radiation Oncology, The Ohio State University, Columbus, OH, USA; c Southern Illinois University School of Medicine, Springfield, IL, USA; d College of Optometry, The Ohio State University, Columbus, OH, USA

**Keywords:** Cataract, Lens epithelium, PDGFRβ, TGFβ, STAT5, IGF1

## Abstract

**Introduction:**

Cataracts are the world’s leading cause of reversible blindness. Although cataract formation is commonly initiated by lens fiber cell defects, cataractogenesis can be characterized by aberrant proliferation and migration of lens epithelial cells. Subsequent overproduction of extracellular matrix components such as fibronectin and collagen by epithelial cells is associated with fibrosis of the lens. Little is known about the role of platelet-derived growth factor receptor β (PDGFRβ) in lens fibrosis. Here, we investigated mice with a conditional knock-in of PDGFRβ hyperactivation using a *Fsp1*, also known as S100A4, promoter (*Fsp1-cre;Pdgfrb*^+*/D849V*^), which consistently develop cataracts at a young age.

**Methods:**

Lenses from *Fsp1-cre;Pdgfrb*^+*/D849V*^ mice and age-matched controls were dissected and visualized via microscopy from 9 to 15 weeks. Early transcriptional changes of the lenses were investigated between 10 and 12 day old *Fsp1-cre;Pdgfrb*^+*/D849V*^ and control mice via RNA sequencing followed by gene set enrichment analysis. Confirmation of RNA sequencing results and mechanistic investigation of PDGFRβ-induced cataractogenesis were determined in lenses isolated from 15-week-old *Fsp1-cre;Pdgfrb*^+*/D849V*^ and control mice.

**Results:**

Gross examination of cataractous lenses from *Fsp1-cre;Pdgfrb*^+*/D849V*^ mice revealed complete opacification by 15 weeks of age compared to no opacification in age-matched controls. Structural changes in the anterior, equatorial, and posterior lens were observed in histology. RNA sequencing revealed significant enrichment of gene sets related to extracellular matrix deposition and reorganization. Mechanistic investigation revealed major roles for TGFβ, Wnt/β-catenin, SOCS2, and STAT5-IGF1 signaling axes in PDGFRβ-induced cataract formation.

**Conclusion:**

PDGFRβ promoted cataractogenesis by modulating pro-fibrotic extracellular matrix changes, likely through TGFβ, Wnt/β-catenin, SOCS2, and the STAT5-IGF1 pathways. Future experiments will delineate the precise role of the STAT5-IGF1 signaling pathway in PDGFRβ-mediated fibrosis and the interplay between PDGFRβ and TGFβ in the lens and whether this signaling is targetable to modulate cataractogenesis.

## 1. Introduction

Cataracts, or opacification of the lens, are the world’s leading cause of reversible blindness (Lam et al., 2015). While cataract surgery, in which the lens is removed and replaced with a new intraocular lens, is a well-established treatment, 20–40 % of cataract surgery patients develop secondary opacification, referred to as posterior capsule opacification (PCO), within 2–5 years of surgery (Awasthi et al., 2009). In advanced cataracts, subcapsular cataract, and PCO, aberrant proliferation and migration of lens epithelial cells (LECs) is driven by epithelial-mesenchymal transition (EMT) (Lovicu et al., 2016). Stimulated LECs that undergo EMT can subsequently overproduce extracellular matrix (ECM) components, such as fibronectin and collagen, inducing fibrosis in the lens (Menko et al., 2020). In the lens, transforming growth factor-beta (TGFβ) is arguably the most well-characterized inducer of EMT and fibrosis, with both TGFβ1 and TGFβ2 isoforms present in the lens (Katsuno et al., 2013; Tan et al., 2015). Platelet-derived growth factors (PDGFs) and their receptors represent another signaling pathway well-known to directly regulate ECM deposition and reorganization, which itself can lead to EMT (Heldin and Westermark, 1999) and fibrosis (Klinkhammer et al., 2018). Within the lens, PDGFs and their receptors are broadly known to play important roles in lens epithelial migration and proliferation, and can potentiate growth factor induced lens fiber differentiation.

Platelet-derived growth factor receptor beta (PDGFRβ) is a receptor tyrosine kinase activated preferentially by ligands platelet-derived growth factor-B (PDGFB) and -D (PDGFD), and this signaling pathway is known to broadly modulate cell migration and proliferation during development and normal physiologic processes (Heldin and Westermark, 1999; Wang et al., 2012). In the eye, tightly regulated PDGFRβ signaling mediates development and proliferation of the lens. The receptor, PDGFRβ, is expressed in the human and rat lens epithelium, and within the hyaloid vasculature of the developing eye, which feeds the lens and then regresses during ocular development (Bozanic et al., 2006; Chen et al., 2019; Ray et al., 2005). Several studies have shown that PDGFB-PDGFRβ regulates the growth, proliferation, and migration of LECs via various mechanisms such as PI3K/AKT, ERK1/2, and JNK activation (Xiong et al., 2010).

PDGFB has also been identified in the retina and vitreous humor of human cataract patients (Zhou et al., 2023), and PDGFD is expressed in newborn rat eyes within the iris, ciliary body, and retina, and the protein has also been detected in bovine aqueous humor (Ray et al., 2005). PDGFD is mitogenic in the lens and PDGFB modulates intracellular calcium homeostasis in LECs (Knorr et al., 1993).

Beyond its role in normal development, dysregulated PDGFRβ signaling has been implicated in fibrotic pathologies of the eye. For example, in proliferative vitreoretinopathy, a fibrotic disease of the retina, PDGFRβ stimulation activates the PI3K-Akt protein kinase B (Akt) pathway (Yang et al., 2020). It is well known that Akt signaling promotes a fibrotic phenotype in many tissue types, including the lens, resulting in EMT and increased expression of ECM proteins such as fibronectin, collagen, and laminin (Menko and Walker, 2022). Neutralization of PDGFRβ in mice with induced choroidal neovascularization had reduced subretinal fibrosis and collagen deposition (Liu et al., 2020). Yet, despite the world-wide prevalence of cataracts and understanding of PDGFRβ-induced fibrosis, there is currently little known regarding the role of PDGFRβ in fibrosis of the lens specifically, a knowledge gap we aim to fill with this study.

Herein, mice with a conditional knock-in of PDGFRβ hyperactivation driven by the *Fsp1*/*S100A4* promoter (*Fsp1-cre;Pdgfrb*^+*/D849V*^) were observed at a young age to develop cataracts, which were strikingly absent in age-matched controls. Based on this observation, we hypothesized that PDGFRβ activity plays a principal role in cataractogenesis by mediating fibrosis of the lens. In this report, we describe the morphologic and transcriptomic changes in the lenses of PDGFRβ hyperactive *Fsp1-cre;Pdgfrb*^+*/D849V*^ mice and describe possible mechanisms for PDGFRβ-induced cataract formation.

## 2. Methods

### 2.1. Transgenic mice and genotyping

Animal use was directed under the Ohio State University Institutional Animal Care and Use Committee (IACUC) Protocol 2007A0120 (principal investigator: G.M. Sizemore). Conditional *Pdgfrb*^+*/D849V*^ knock-in mice originally mixed C57Bl6/129S4 (Olson and Soriano, 2011; Thies et al., 2021) (Jackson Laboratories, Strain #018435) were crossed with male FVB/N *Fsp1-cre* mice (Trimboli et al., 2009) and bred into the FVB/N strain at least 9 generations. These mice were further crossed with the *Rosa-LSL-tdTomato* reporter allele at generation 8 (Jackson Laboratories, Strain #007905), which had also been back crossed into the FVB/N strain prior to introduction into this *Fsp1-cre; Pdgfrb*^+*/D849V*^ line. For maintenance of the line, male *Fsp1-cre*;*tdTomato* or *Fsp1-cre;tdTomato;Pdgfrb*^+*/D849V*^ mice were bred with *tdTomato; Pdgfrb*^+*/D849V*^ female mice. Litters maintained the expected Mendelian genetic ratio of 3 control mice (*Fsp1-cre;Pdgfrb*^+*/*+^, *Pdgfrb*^+*/*+^, and *Pdgfrb*^+*/D849V*^) for every 1 mutant mouse (*Fsp1-cre;Pdgfrb*^+*/D849V*^). DNA was isolated from mouse ear or tail clips and genotyped by PCR analysis using the following primer sets (5′ to 3’): *Cre*, GCGGTCTGGCAGTAAAAACTATC (forward) and GTGAAACAGCATTGCTGTCACTT (reverse); *Pdgfrb*^+*/D849V*^, GGGCTTCCAGGAGTGATACC (Common), CCAGCTGGACTGAAGAGGAG (WT reverse), and CCGAGCAGGTCAGAACAAAG (mutant reverse); *tdTomato*, AAGGGAGCTGCAGTGGAGTA (WT forward), CCGAAAATCTGTGGGAAGTC (WT reverse), CTGTTCCTGTACGGCATGG (transgene forward), GGCATTAAAGCAGCGTATCC (transgene reverse).

### 2.2. Lens dissection

*Fsp1-cre;Pdgfrb*^+*/D849V*^ mice and age matched controls were euthanized and eyes immediately enucleated at 10–15 weeks of age. To obtain lenses, eyes were immediately dissected in room temperature Phosphate-Buffered Saline (PBS) (Corning). Ciliary body and suspensory ligaments were manually removed. Lenses were then washed in fresh PBS and images were taken with a Leica MZ10 F Stereomicroscope. To visualize the cataract, lenses were imaged over a transmission electron microscopy grid.

### 2.3. Hematoxylin & eosin staining and lens visualization

For hematoxylin and eosin staining (H&E), all samples were formalin fixed and embedded in paraffin by the OSU Comprehensive Cancer Center’s Comparative Pathology and Digital Imaging Shared Resource. Four μm thick sections were cut and stained with H&E. Images were taken with a Nikon Eclipse 80i microscope. For visualization of tdTomato positive cells, whole eyes were fixed in 4 % PFA for 24 h, washed with PBS, cryoprotected in 30 % sucrose for 48 h, embedded in OCT and flash frozen on dry ice. Eyes were cryo-sectioned in 15 μm sagittal sections via a Leica CM1950 cryostat. Sections were fixed in 4 % PFA for 15 min, washed 3x in 1x PBS, and permeabilized in 1 % PBST for 30 min. Following permeabilization, eyes were incubated for 3 min with Hoescht (ThermoFisher), washed 2x in 1x PBS, and mounted. Slides were imaged on an EVOS M7000 and tdTomato expression visualized with the txRed laser.

### 2.4. RNA sequencing

Mice between the ages of 10–12 days were euthanized, followed immediately by enucleation and lens dissection in room temperature PBS in an RNAse-free environment. PBS was wicked off the lenses using a Kimwipe and lenses were then immediately placed into a micro-centrifuge tube and flash frozen on dry ice. Lenses were pooled from multiple mice to ensure adequate RNA for sequencing. For control mice, pooled mice included *Fsp1-cre;Pdgfrb*^+*/*+^, *Pdgfrb*^+*/*+^, and *Pdgfrb*^+*/D849V*^ genotypes. RNA extraction, sequencing, differential gene expression, and gene set enrichment analysis was performed by Novogene and revisualized using Graphpad Prism v10 and RStudio [v 4.2.2 (R-Core-Team, 2021)] via EnhancedVolcano [v1.16.0 (Kevin Blighe and Myles, 2020)]. The heatmap was created using pheatmap [v1.0.13 (Kolde, 2019)] with clustering defined by the standard z-score scaling. Read alignment was performed via Hisat2 [v2.0.5 (Kim et al., 2019)], quantification via featureCounts [v1.5.0-p3 (Liao et al., 2014)], differential expression analysis via DESeq2 [v1.20.0 (Love et al., 2014)] and enrichment analysis via clusterProfiler [v3.8.1; G (Yu et al., 2012)] using KEGG, GO, and Reactome terms.

### 2.5. RNA extraction and quantitative RT-PCR

Lenses were isolated from 15-week old *Fsp1-cre;Pdgfrb*^+*/D849V*^ and control mice (*Fsp1-cre;Pdgfrb*^+*/*+^) in an RNase free environment and flash frozen on dry-ice. RNA was isolated using TRIzol (Thermo Fisher) as described previously (Han et al., 2024). Isolated RNA was then converted to cDNA following standard procedures using the Maxima First Strand cDNA Synthesis Kit (Thermo Fisher, Catalog #K1641). Taqman assays (Thermo Fisher) were used to perform quantitative RT-PCR for *Lama2* (Mm00550083_m1), *Grik1* (Mm00446882_m1), *Socs2* (Mm01247834 _m1), *Stat5a* (Mm00839861_m1), *Igf1* (Mm00439560_m1), *Tgfb1* (Mm01178820_m1), *Wnt5b* (Mm01183986_m1), *Wnt7b* (Mm01301717_m1), *Wnt8a* (Mm00436822_m1), and *Gapdh* (GAPDH DQ, #4352661). RT-qPCR was performed using PerfeCTa FastMix II ROX (Quanta Biosciences, Catalog #95119–05K) according to manufacturer’s instructions. Relative quantification of each gene was calculated and normalized to *Gapdh* expression.

### 2.6. Western blots

Lenses were isolated from 15-week old *Fsp1-cre;Pdgfrb*^+*/D849V*^ and control mice (*Fsp1-cre;Pdgfrb*^+*/*+^) and flash frozen on dry-ice. Protein was isolated and western blots performed as previously described (Sizemore et al., 2007). Membranes were incubated in primary antibody purchased from Cell Signaling (STAT5 #94205, P-STAT5 #9359, SMAD2 #5339, TGFβ #3711, β-catenin #9562), Abcam (P-SMAD2 #188334), or Millipore Sigma (α-tubulin, #T6199) overnight at 4 °C or 1 h at room temperature (α-tubulin). Membranes were incubated with HRP-conjugated secondary antibody for 1 h at room temperature and bands were visualized with Immobilon ECL reagent (Millipore) using an Amersham Imager 600 instrument. Quantification of protein expression by band densitometry was performed in ImageJ (Schroeder et al., 2021).

### 2.7. Statistical analysis

Statistical analyses were conducted in GraphPad Prism v10.3.1. All data were tested for normality via D’Agostino-Pearson omnibus, Shapiro-Wilk, and Kolmogorov-Smirnov normality tests and were considered normal after passing any of the three tests. Statistical comparisons of two groups were completed via unpaired Student’s t-test with Welch’s correction for unequal variance when applicable. Statistical significance was set as *P* ≤ 0.05.

## 3. Results

### 3.1. Fsp1-driven PDGFRβ hyperactivity causes progressive cataractogenesis

The *Fsp1-cre;Pdgfrb*^+*/D849V*^ model was developed previously by our team to study hyperactive PDGFRβ signaling in the breast tumor microenvironment through knock-in of mutant PDGFRβ-D849V into the endogenous locus (Thies et al., 2021). During the course of these prior studies, an ocular phenotype was observed in the *Fsp1-cre;Pdgfrb*^+*/D849V*^ mice. Of note, neither the *Fsp1-cre* or the *Pdgfrb*^+*/D849V*^ alleles are lens-specific. Aside from this ocular phenotype, which we describe herein, we observed no additional overt differences in development or normal physiology in these animals.

**Fig. 1.**
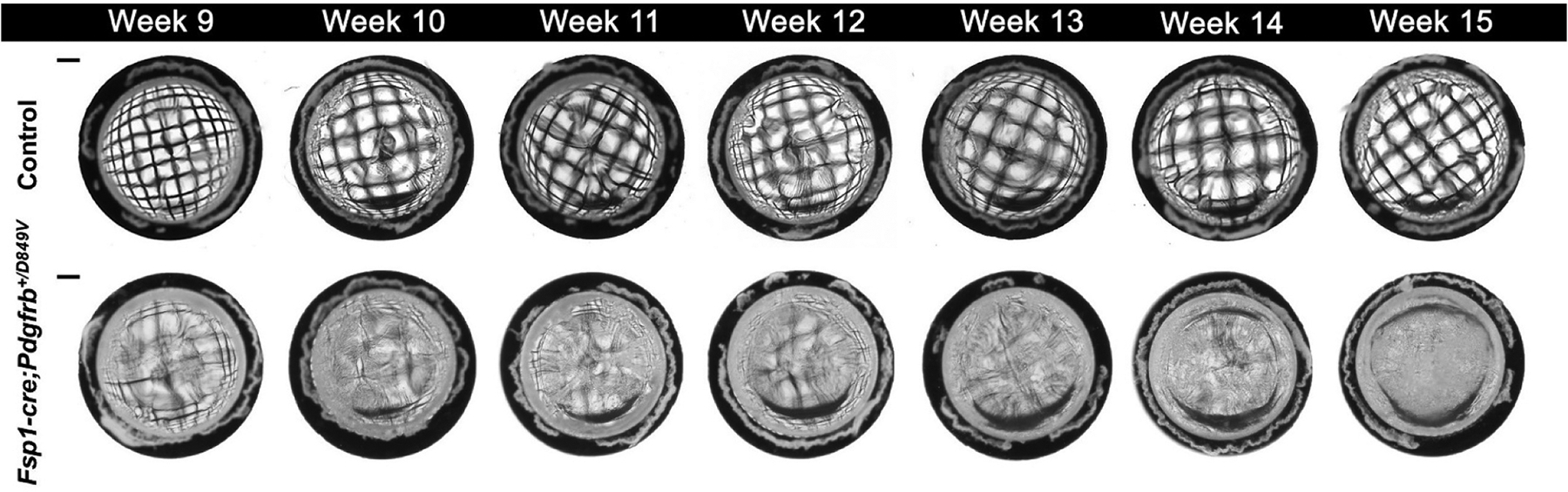
Progressive cataractogenesis in mice with *Fsp1*-driven PDGFRβ hyperactivity. Representative lenses from *Fsp1-cre;Pdgfrb*^+/*D849V*^ mice and age-matched controls from 9 to 15 weeks of age were imaged over a transmission electron grid and evaluated for cataract. Control, 9wk n = 3; 10wk n = 7; 11wk n = 6; 12wk n = 4; 13wk n = 8; 14wk n = 6; 15wk n = 3. *Fsp1-cre;Pdgfrb*^+/*D849V*^, 9wk n = 3; 10wk n = 6; 11wk n = 3; 12wk n = 3; 13wk n = 3; 14wk n = 3; 15wk n = 3. Scale bars = 0.25 mm.

**Fig. 2.**
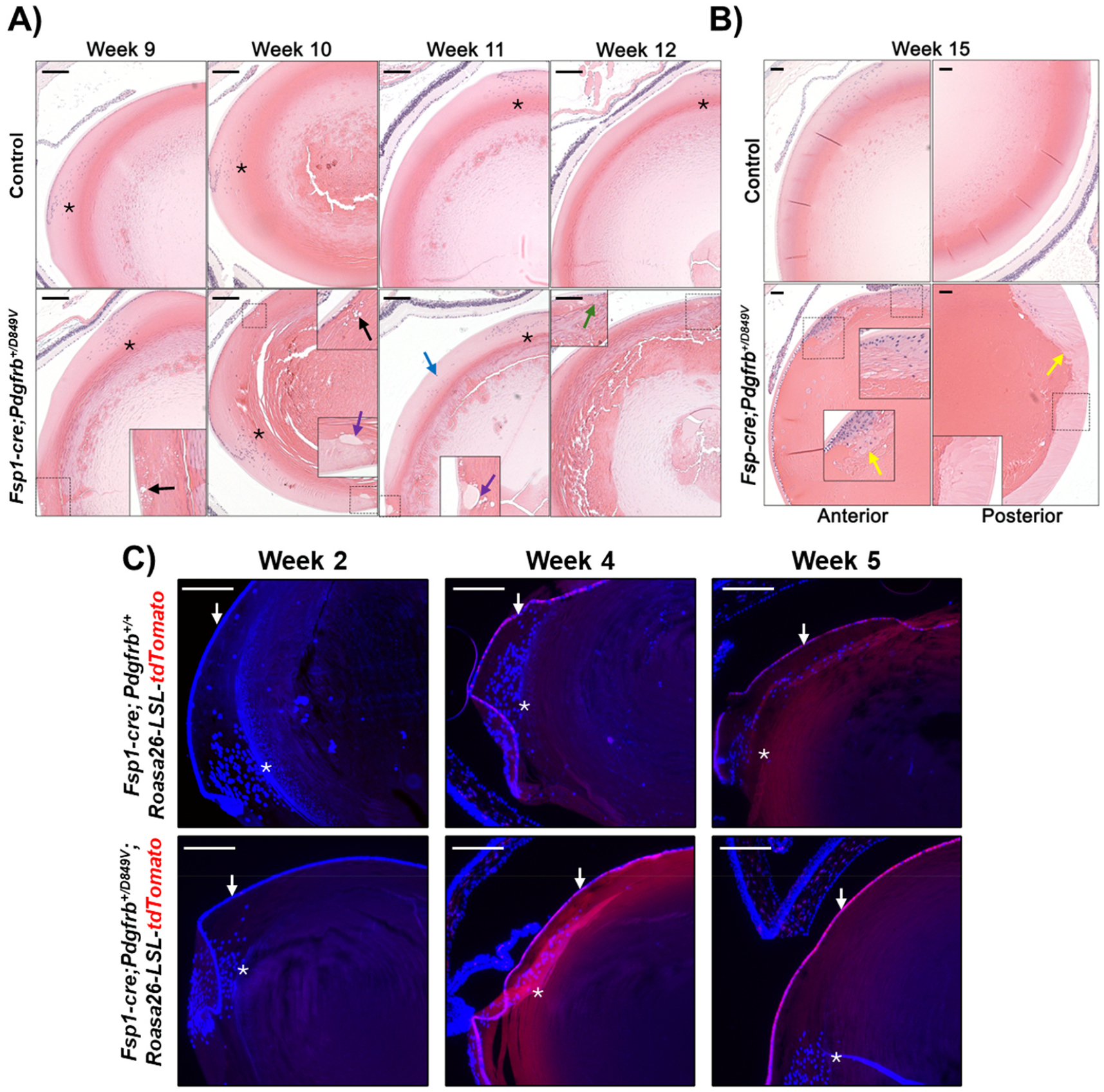
Anterior and posterior subcapsular cataracts with progressive structural cortical changes in young mice with *Fsp1*-driven PDGFRβ hyperactivity. **A)** H&E staining of lenses from *Fsp1-cre;Pdgfrb*^+*/D849V*^ mice versus age-matched controls at weeks 9 through 12. Control, 9wk n = 3; 10wk n = 7; 11wk n = 7; 12wk n = 6. *Fsp1-cre;Pdgfrb*^+*/D849V*^, 9wk n = 3; 10wk n = 4; 11wk n = 3, 12wk n = 3. Vacuole formation (black arrows), proteinaceous globules (purple arrows), retention of cell nuclei beyond the transition zone (blue arrow), and disorganization of the cortex at the region of suture formation (green arrow) were observed. **B)** The anterior and posterior lens from 15-week-old *Fsp1-cre;Pdgfrb*^+*/D849V*^ and control mice (control, n = 4. *Fsp1-cre;Pdgfrb*^+*/D849V*^, n = 4) indicate abnormal cortical differentiation and protein globules in *Fsp1-cre;Pdgfrb*^+*/D849V*^ mice (yellow arrows). **C)** tdTomato positivity (red) in lenses from *Fsp1-cre;Pdgfrb*^+*/D849V*^ mice versus age-matched controls at weeks 2, 4 and 5. Control, 2wk n = 1; 4wk n = 2; 5wk n = 2. *Fsp1-cre;Pdgfrb*^+*/D849V*^, 2wk n = 3; 4wk n = 2; 5wk n = 1. White arrow indicates lens epithelial cells. For A–C, the asterisk indicates the location of the lens equator and scale bars = 200 μm.

To study the ocular phenotype, lenses were collected from *Fsp1-cre; Pdgfrb*^+*/D849V*^ and age-matched control mice (includes *Fsp1-cre;Pdgfrb*^+*/*+^, *Pdgfrb*^+*/*+^, and *Pdgfrb*^+*/D849V*^ genotypes for all studies unless otherwise indicated) and imaged over a transmission electron grid to visualize lens transparency and cataract development over time. *Fsp1-cre;Pdgfrb*^+*/D849V*^ mice showed rapid and complete opacification by 15 weeks of age with obvious progression occurring from 9 to 15 weeks, compared to transparent age matched controls at all time points (Fig. 1). Of note, there are inherent regions of discontinuity within the normal crystalline lens due to changes in the refractive index and suture development (Fagerholm et al., 1990; Koretz et al., 1994). Similar to slit lamp biomicroscopy, sutures and zones of discontinuity can be observed in a normal lens depending on the plane of focus and the light conditions used, which explains some of the aberrations observed in the control lenses. Importantly, transparency is not lost in these control lenses and, as seen in Fig. 2, histology confirms the normal morphologic appearance of the control lenses.

Histologically, cells in the *Fsp1-cre;Pdgfrb*^+*/D849V*^ anterior, equatorial, and posterior lens demonstrated widespread disruption in organization, consistent with tissue opacification (Fig. 2). These lenses exhibit disorganized proliferation and differentiation of cells anteriorly and posteriorly, which progressed with time (Fig. 2A and B). Histology further revealed vacuole formation within the lens beginning first at the anterior and posterior poles (Fig. 2A, black arrows), the presence of proteinaceous globules (Fig. 2A, purple arrows), and progressing to pronounced dysregulation of lens cortex differentiation (Fig. 2B). Disruption and proliferation of the normally-quiescent lens epithelial monolayer can be seen beginning at approximately week 10 in *Fsp1-cre; Pdgfrb*^+*/D849V*^ mice. Additionally, disorganized differentiation is apparent starting in the week 11 *Fsp1-cre;Pdgfrb*^+*/D849V*^ lenses, with nuclei of cortical fiber cells still present (Fig. 2A, blue arrow) and cells located more posteriorly, compared to controls. By week 12, cortical cell architecture and arrangement, close to the lens sutures, are clearly abnormal (Fig. 2A, green arrow). By week 15, clear loss of quiescence in the epithelial monolayer is present (Fig. 2B). Aggregations of proliferating cells are seen in the anterior and posterior lens accompanied by globular lens fibers (Fig. 2B, yellow arrows), unlike the expected regular, concentric ring-shaped lens fibers in controls. Examination of the transition zone in *Fsp1-cre;Pdgfrb*^+*/D849V*^ lenses shows differentiating cortical fibers with distinctly pale cytoplasm and abnormal width, and abnormal retention of the fiber nucleus and organelles. These dramatic histologic changes within the lens correspond to the loss in tissue transparency observed in Fig. 1.

Overall, these findings suggest that *Fsp1-cre;Pdgfrb*^+*/D849V*^ mice develop cataract early in life. Importantly, although the phenotype does not appear until week 9, the *Fsp1-cre* transgene is turning on as early as week 4, where robust *Rosa-LSL-tdTomato* reporter activity is observed in the lens epithelium of both control *Fsp1-cre;Rosa-LSL-tdTomato* and *Fsp1-cre;Rosa-LSL-tdTomato;Pdgfrb*^+*/D849V*^ mice (Fig. 2C). Given that the *Pdgfrb*^+*/D849V*^ allele is under control of the endogenous *Pdgfrb* promoter, simply the expression of Cre reported by tdTomato is alone not confirmation that the mutant receptor is expressed. However, we know from the literature that, in humans, *PDGFRB* is expressed within the developing eye in conceptuses of 5–9 postovulatory weeks (Bozanic et al., 2006). Similarly, *Pdgfrb* has been detected in newborn lens epithelial cells and lens fiber cells in mice (Hoang et al., 2014). Thus, it is assumed that *Pdgfrb* expression is similarly turned on *in utero* in these mice and upon induction of the Cre, the mutant PDGFRβ-D849V is expressed.

**Fig. 3.**
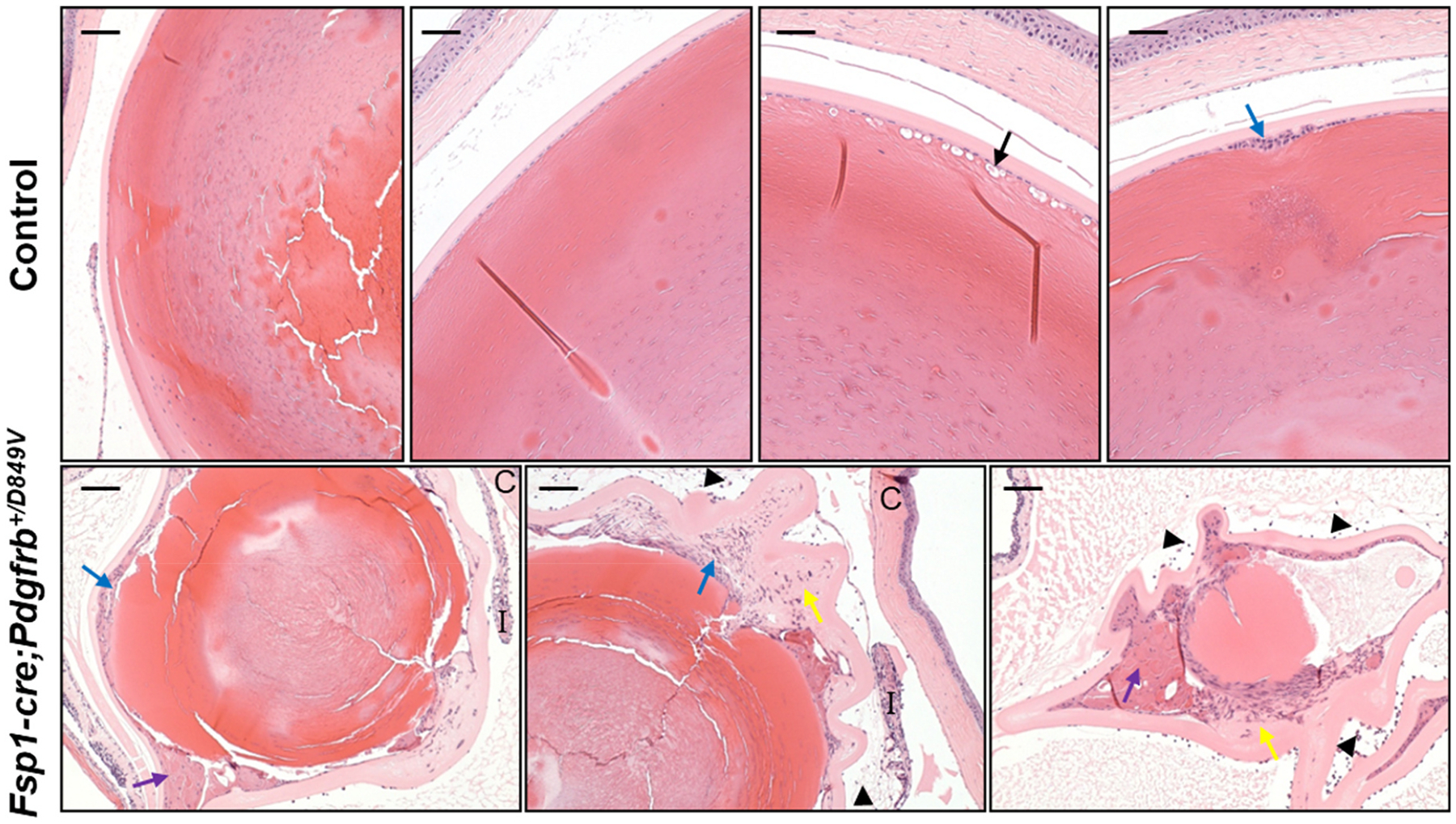
Severe cataract formation, lens-induced uveitis, and advanced fibrous metaplasia in aged mice with *Fsp1*-driven PDGFRβ hyperactivity. H&E staining of lenses from *Fsp1-cre;Pdgfrb*^+*/D849V*^ mice versus age-matched controls aged over one year. Each image is from a biological replicate (control, n = 4. *Fsp1-cre; Pdgfrb*^+*/D849V*^, n = 3). Control mice exhibit minor age-related cataract, such as focal vacuole formation (black arrow) or epithelial proliferation (blue arrow). *Fsp1-cre; Pdgfrb*^+*/D849V*^ mice have substantial anterior and posterior epithelial proliferation (blue arrows) and fibrous metaplasia with pronounced cortical liquefaction (i.e. proteinaceous globules; purple arrows), and deposition of ECM (yellow arrows). Protein debris and uveitis (black arrowheads) can be seen in both the anterior and posterior chambers of the *Fsp1-cre;Pdgfrb*^+*/D849V*^ mice. The iris is indicated by “I” and cornea by “C”. Scale bars = 200 μm.

To evaluate how the observed cataract phenotype worsens over time, mice were aged (>1 year) and evaluated. Not unexpectedly, the *Fsp1-cre;Pdgfrb*^+*/D849V*^ mice exhibit severe cataract formation (Fig. 3, purple arrows), lens-induced uveitis (Fig. 3, black arrowheads), and advanced fibrous metaplasia (Fig. 3, yellow arrows), while age-matched controls demonstrate normal, more subtle, age-related lens changes (Fig. 3, black arrow). Taken together, these findings suggest a phenotype that begins early in life that continues to worsen with time and support an important role for PDGFRβ in the development of progressive cataractogenesis.

### 3.2. Fsp1-driven PDGFRβ hyperactivity causes transcriptional changes in the lens indicative of fibrosis

**Fig. 4.**
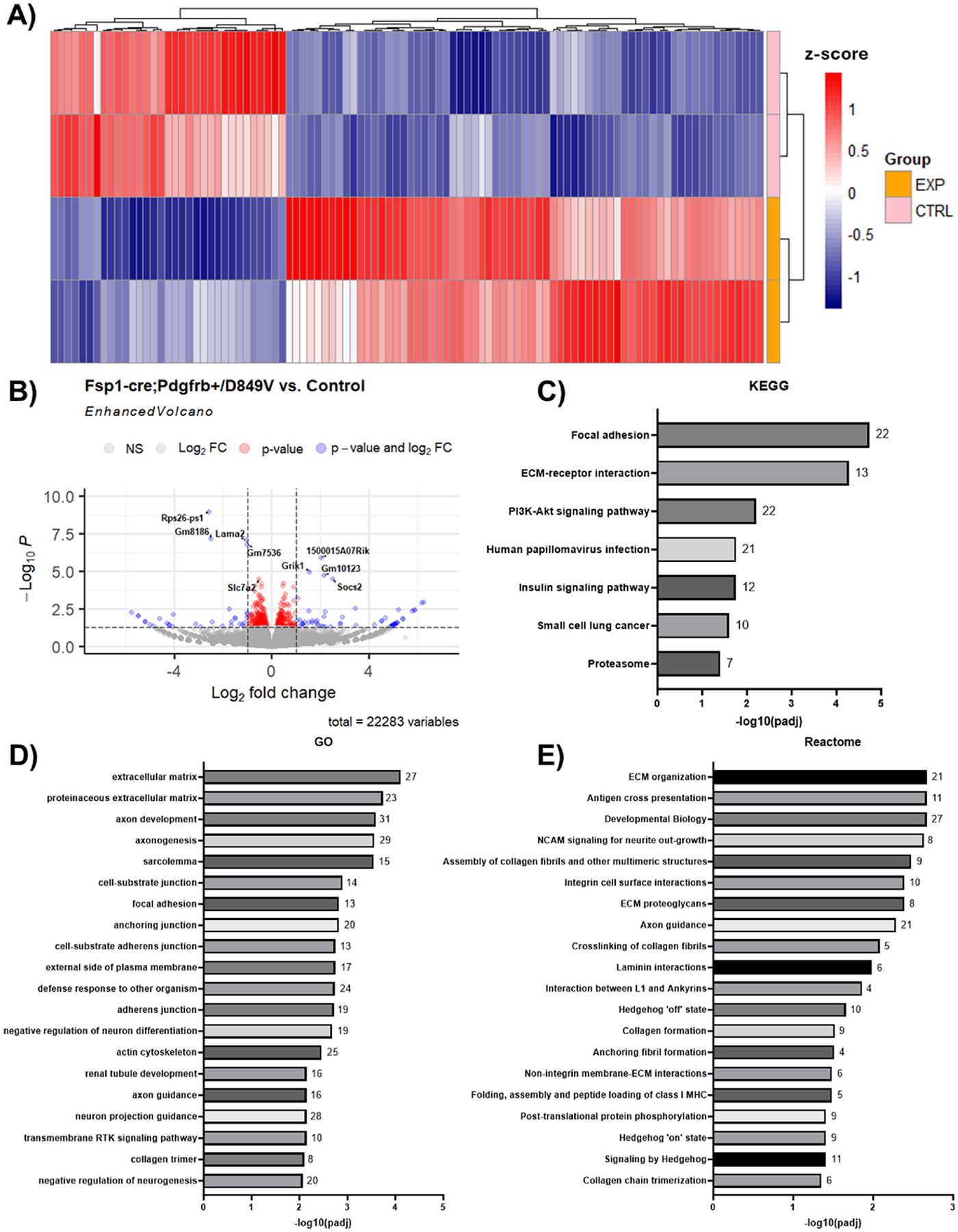
RNA sequencing of lenses from 10–12 day-old *Fsp1-cre;Pdgfrb*^+/D849V^ mice and age-matched controls reveal distinct transcriptomic changes and fibrosis-related pathway enrichment. **A)** Heatmap showing overall transcriptomic profile differences between lenses from *Fsp1-cre;Pdgfrb*^+*/D849V*^ and control mice. **B)** Volcano plot showing differential expression analysis with the significantly differentially expressed genes labeled (adjusted p-value <0.05). **C-E)** Enrichment analysis showing gene sets enriched in *Fsp1-cre;Pdgfrb*^+*/D849V*^ mice compared to controls in KEGG **(C)**, GO **(D)**, and Reactome **(E)** gene set databases where numbers at the end of each bar represent enriched gene count (n = 12/group).

To understand the early transcriptomic changes responsible for the formation of cataracts in the presence of *Fsp1*-driven PDGFRβ hyperactivity, we performed RNA sequencing of lenses from 10 to 12 day old *Fsp1-cre;Pdgfrb*^+*/D849V*^ and control mice. These analyses revealed distinct transcriptomic changes via heatmap clustering, with experimental groups clustering tightly together (Fig. 4A). Differential gene expression analysis revealed nine significantly differentially expressed genes that included *Rps26-os1, Lama2, Gm8186, Gm7536, Gm7536, 1500015A07Rik, Grik1, Gm10123, Socs2,* and *Slc7a2* (Fig. 4B–Table 1). Of note, *Pdgfrb* is not differentially expressed due to the nature of the mouse model, which knocks in the mutant *Pdgfrb*^+*/D849V*^ under the control of the endogenous *Pdgfrb* promoter. Further bioinformatic investigation using gene set enrichment analysis (GSEA) via KEGG, GO, and Reactome gene set databases was also conducted (Fig. 4C–E). Interestingly, we observed significant enrichment of ECM-related gene sets across each database. For example, we detected significant enrichment in KEGG gene sets representing “Focal adhesion” and “ECM-receptor interaction” (Fig. 4C), in GO gene sets representing “extracellular matrix”, “proteinaceous extracellular matrix”, and “focal adhesion” (Fig. 4D), and in Reactome gene sets representing “ECM organization”, “Assembly of collagen fibrils and other multimeric structures”, “ECM proteoglycans”, and “crosslinking of collagen fibrils” (Fig. 4E). Together, these changes all indicate significant alteration in ECM deposition and organization in our mutant mice. Of note, we also observed enrichment in “PI3K-Akt signaling pathway” and “transmembrane RTK signaling pathway” gene sets, which was to be expected given the forced PDGFRβ hyperactivity. Lastly, in the Reactome terms analysis, we also observed enrichment in “Hedgehog ‘off’ state”, “Hedgehog ‘on’ state”, and “Signaling by Hedgehog”, consistent with the known function of Lama2, one of the differentially expressed genes, in mesenchymal stem cell fate commitment (Zhu et al., 2020). Together, these results suggest that the observed PDGFRβ-mediated fibrosis in the lens could be due to the induction of ECM protein changes that may be a result of aberrant EMT. However, from this analysis alone, it is not possible to know which of these pathways are dysregulated first leading directly to the observed phenotype in the eye.

### 3.3. Fsp1-driven PDGFRβ hyperactivity induces pro-cataractous TGFβ and STAT5-IGF1 signaling

**Fig. 5.**
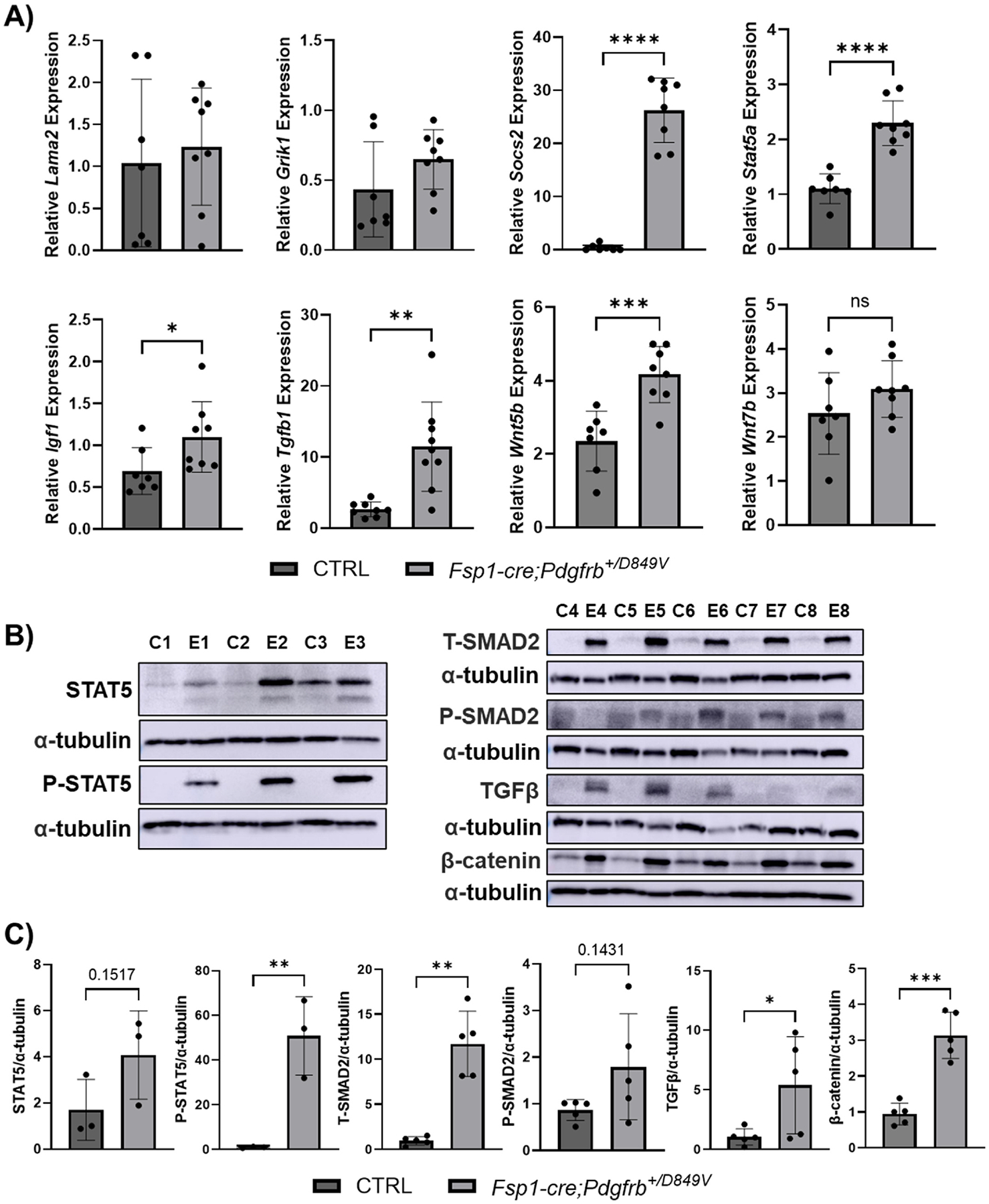
*Fsp1*-driven PDGFRβ hyperactive signaling induces pro-cataractous TGFβ-SMAD2, Wnt/β-catenin, and STAT5-IGF1 signaling. **A)** Relative quantification of *Lama2, Grik1, Socs2, Stat5a*, *Igf1*, *Wnt5b*, and *Wnt7b* in lenses isolated from 15-week-old mice by qRT-PCR (control, n = 7. *Fsp1-cre;Pdgfrb*^+*/D849V*^, n = 8). **B)** Western blot of lenses isolated from 15-week-old control (C) or *Fsp1-cre;Pdgfrb*^+*/D849V*^ experimental (E) mice and **C)** quantification (n = 3/group (left) or 5/group (right)). * <0.05, ** <0.01, **** <0.0001.

In order to validate the RNA sequencing results in animals with advanced cataract formation, we then isolated lenses from 15-week-old *Fsp1-cre;Pdgfrb*^+*/D849V*^ and control mice for further investigation. Quantitative real-time PCR revealed that *Lama2* and *Grik1* expression were not significantly different at 15 weeks, but a significant upregulation of *Socs2* was observed at this time point (Fig. 5A). This drastic upregulation of *Socs2,* along with previous literature identifying PDGFRβ induction of STAT5, pointed towards the STAT5-IGF1 signaling axis, which SOCS2 can both positively and negatively regulate depending on expression levels (Greenhalgh et al., 2005; Valgeirsdottir et al., 1998). As such, we evaluated both *Igf1* and *Stat5a* mRNA expression and STAT5 activation in isolated lenses. Importantly, *Fsp1-cre;Pdgfrb*^+*/D849V*^ lenses had significantly upregulated *Igf1* and *Stat5a* mRNA, and although we did not observe a significant difference in 10–12 day old mice, lenses from 15-week old *Fsp1-cre;Pdgfrb*^+*/D849V*^ mice had significant upregulation of the pro-fibrotic factor *Tgfb1* (Fig. 5A). TGFβ has previously been shown to induce *Wnt* family gene expression during cataract formation (Chong et al., 2009). Interestingly, we observed a significant upregulation of *Wnt5b,* no change in *Wnt7b* (Fig. 5A) and undetectable expression of *Wnt8a* (data not shown). Western blot analysis confirmed *Fsp1-cre;Pdgfrb*^+*/D849V*^ mice (denoted as E1–8) had significantly higher levels of STAT5 signaling, as indicated by total and phosphorylated STAT5 (Fig. 5B and C). TGFβ protein level increases were confirmed, as well as increased signaling through SMAD2 and increased β-catenin (Fig. 5B and C). Taken together, these results indicate that early pro-cataract changes appear to be mediated by EMT and ECM reorganization, possibly induced by Lama2 and Socs2, with prolonged activation of the TGFβ-SMAD2, Wnt/β-catenin, and STAT5-IGF1 pathways modulating the progression to mature cataract in early adult mice.

## 4. Discussion

Here, we report a timeline of gross cataractous changes in the lenses of mice with *Fsp1*-driven hyperactivation of PDGFRβ. The lenses were analyzed through RNA sequencing and subsequent gene expression enrichment analysis identified pro-cataractous transcriptomic changes. These RNA sequencing results were further confirmed by qRT-PCR and western blot analysis, expanding the mechanistic understanding of how PDGFRβ signaling induces cataracts.

The observed cataractogenesis in *Fsp1-cre;Pdgfrb*^+*/D849V*^ mice was profound. Imaging of lenses from mice 9–15 weeks of age revealed progressive wrinkling and opacification of *Fsp1-cre;Pdgfrb*^+*/D849V*^ lenses compared with age matched controls. In wild-type mice, subcapsular or cortical cataracts may appear after 12 months of age, with age-related cataracts typically beginning in the nucleus and cortex of the lens (Age-Related Eye Disease Study Research, G., 2001; Cheng et al., 2019). The lens grows continuously throughout life due to the addition of newly differentiated fibers arranged in concentric growth shells. Although not completely elucidated, the etiology of age-related cataracts likely involves a combination of compression and stiffening due to increased density of the lens, as well as loss of soluble proteins (Asbell et al., 2005; Giblin et al., 1982; McFall-Ngai et al., 1985; Truscott, 2005). Unlike age-related cataracts, lenses from *Fsp1-cre;Pdgfrb*^+*/D849V*^ mice developed cataracts as early as 9 weeks, with lenses from mice aged 9–12 weeks showing anterior and posterior subcapsular cataract, as well as cortical cataract, denoting an early pathologic mechanism that is unique to PDGFRβ-induced cataractogenesis. By 15 weeks of age, mature cataracts are observed in *Fsp1-cre;Pdgfrb*^+*/D849V*^ mice where significant posterior subcapsular cataract and ultrastructural cortical changes are seen. Similar to the work presented by others, we show that cataract progression can occur in a non-uniform manner with some areas of the lens displaying disorganization and other areas of the lens appearing relatively normal (Kwok and Coroneo, 2000; Vrensen and Willekens, 1990). Lenses may develop focal disruption within the epithelial monolayer or at varying depths within affected fibers, leading to small areas of opacification. Progression of the disrupted fibers can expand along the circumference, expanding to disruptions with adjacent fibers, leading to variable rates of opacification growth (Brown et al., 1993; Martin et al., 2022).

Subcapsular cataracts can occur due to retinal degeneration, trauma, or following phacoemulsification, as a result of aberrant LEC migration and proliferation (Andersson et al., 2003; Fishman et al., 1985; Heckenlively, 1982; Wormstone et al., 2009). There are also reports of subcapsular cataracts with abnormal lens fiber growth resulting in the failure to form typical sutures in successive growth shells (Joy and Al-Ghoul, 2014). Interestingly, within the lens, signaling through the PI3K-Akt pathway, which is downstream of PDGFRβ, balances proliferation versus differentiation, with lens fibers expressing Notch ligand Jag1 enhanced by PI3K signaling (Li et al., 2019). PDGFRβ is expressed both in epithelial and fiber cells of newborn mice (Hoang et al., 2014). Considering that lens growth, epithelial proliferation, and fiber differentiation is greatest during development, it is possible that hyperactive PDGFRβ signaling contributes to the early and rapid structural changes resulting in the observed subcapsular cataract in early life. Phenotypes of subcapsular cataracts from *Fsp1-cre;Pdgfrb*^+*/D849V*^ lenses also showed marked proliferation and aggregation of basophilic, mitotic figures outside of the equator, where the normally quiescent epithelial monolayer should be. Due to PDGFRβ′s broad role as a mitogen across many organ systems and PDGFRβ being present in the lens epithelium, our data suggests a proliferative or fibrotic mechanism of cataractogenesis involving EMT may drive this phenotype. Lastly, because all *Fsp1-cre; Pdgfrb*^+*/D849V*^ mice consistently developed cataracts with locations and patterns distinct from age-related cataracts, we can suggest that the phenotype and mechanism of cataract formation is unique to PDGFRβ-mediated fibrosis.

To investigate how *Fsp1*-driven hyperactivity of PDGFRβ is causing the gross cataract formation in these mice, we performed RNA sequencing of the lenses at a young age. This transcriptomic analysis of 10–12 day old mice revealed statistically significant changes in the expression of multiple genes including *Lama2* and *Socs2*, which are known to function in EMT, ECM production, and fibrosis-related pathways, and thus are likely functioning in the early PDGFRβ-mediated cataractogenesis observed in these mice. These changes could account for the observed asymmetry in lens development, prior to obvious cataract formation and loss of transparency. Additional cataractous changes such as cortical lens fiber disruption and vacuole formation are likely secondary to initial perturbations in fibrosis-mediated cataract pathways. Of note, it has been previously established that Cre recombinase expression in the lens can lead to microphthalmia and lens toxicity through modulation of apoptosis related genes (Lam et al., 2019). Control samples utilized for RNA sequencing were pooled from *Fsp1-cre;Pdgfrb*^+*/*+^, *Pdgfrb*^+*/*+^, and *Pdgfrb*^+*/D849V*^ mice. As such, Cre negative samples could have confounded our transcriptomic analysis. However, all qPCR and western blot analyses were conducted utilizing only lenses from *Fsp1-cre;Pdgfrb*^+*/*+^ mice, which confirmed the important transcriptomic changes we identified via RNA sequencing.

*Lama2*, the gene encoding laminin 2, is downregulated in 10–12 day old *Fsp1-cre;Pdgfrb*^+*/D849V*^ lenses, and has been shown to inhibit differentiation of mesenchymal stem cells via Hedgehog signaling in humans through osteo- and adipo-genesis (Zhu et al., 2020). In human patients with LAMA2 deficient muscular dystrophies, extracellular matrix and interstitial fibrosis-related genes were upregulated as early as 20 days of age (Taniguchi et al., 2006), which mimics the highly profound, early phenotype observed herein. In addition, we also observed significant enrichment of Hedgehog related gene sets in *Fsp1-cre;Pdgfrb*^+*/D849V*^ mice. Hedgehog signaling has been shown to directly promote ECM production and fibrosis in mouse models of obstructive nephropathy (Ding et al., 2012). With both downregulation of Lama2 and upregulation of hedgehog signaling promoting fibrosis via modulation of ECM in disease models, we hypothesize that early downregulation of *Lama2* in the lenses of *Fsp1-cre;Pdgfrb*^+*/D849V*^ mice facilitates EMT in LECs through Hedgehog signaling, thereby remodeling lens ECM and inducing fibrosis. In support, our gene set enrichment analysis revealed a significant enrichment in many ECM-related gene sets.

**Fig. 6.**
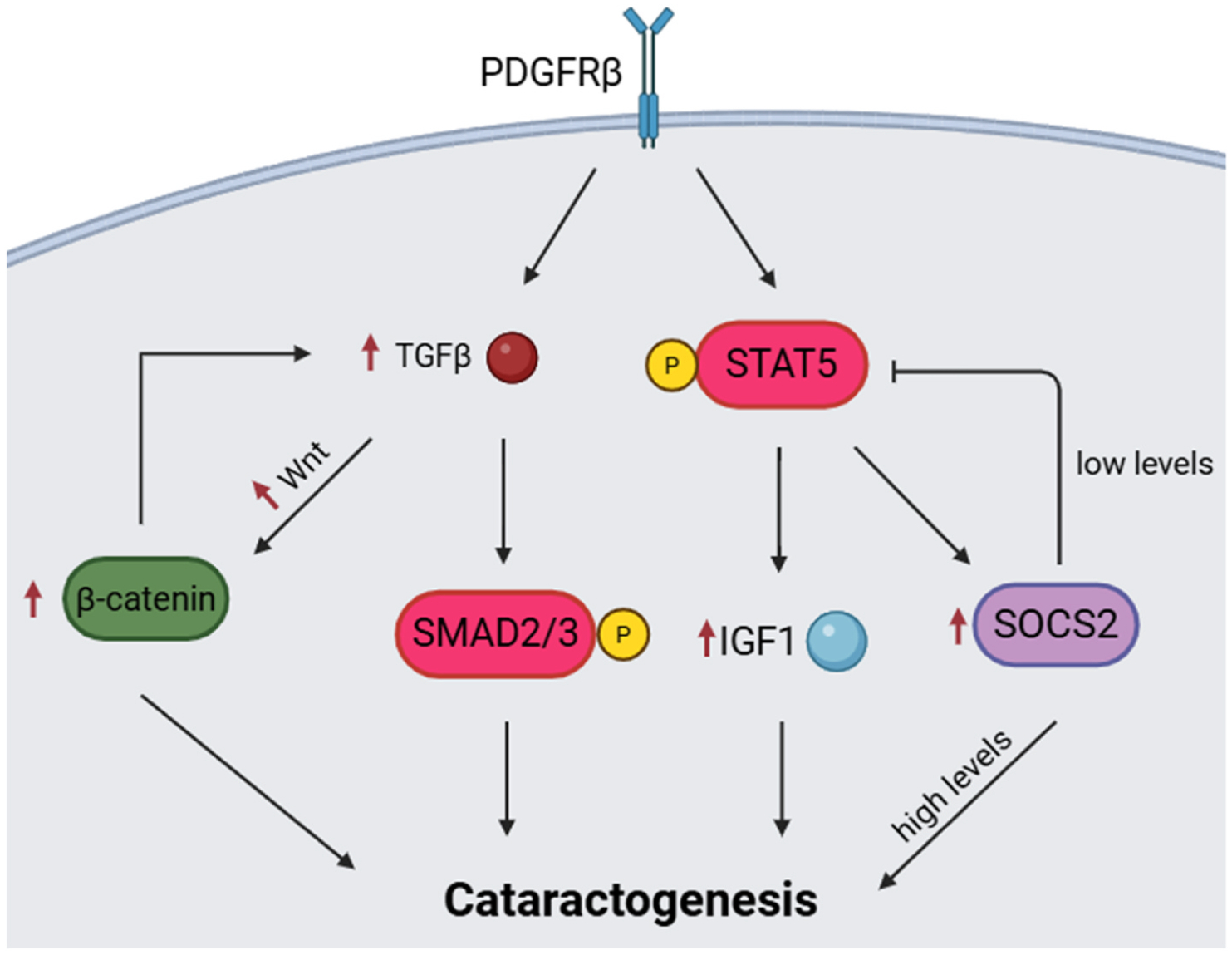
Hypothetical mechanism of PDGFRβ induced cataractogenesis. Hyperactivation of PDGFRβ leads to an upregulation of TGFβ, which leads to increased Wnt/β-catenin and SMAD2 signaling. PDGFRβ signaling also induces increased expression and hyper-phosphorylation of STAT5, which subsequently induces overexpression of IGF1 and SOCS2. Each pathway is predicted to increase cataractogenesis through proliferation, ECM modulation, and fibrosis. Created in BioRender. Banthin, E. (2025) https://BioRender.com/eghtjio.

*Socs2*, the gene encoding suppressor of cytokine signaling 2, was robustly upregulated in 10–12 day and 15-week old *Fsp1-cre;Pdgfrb*^+*/D849V*^ lenses. Socs2 is a known regulator of the STAT5 signaling cascade, where low levels inhibit, and high levels potentiate STAT5-IGF1 signaling (Greenhalgh et al., 2005). STAT5 has previously been shown to be directly induced by PDGFRβ activation (Valgeirsdottir et al., 1998). Interestingly, *Fsp1-cre;Pdgfrb*^+*/D849V*^ lenses had upregulation of both *Igf1* and *Stat5a* mRNA and hyperactivation of STAT5 signaling. Combined, supporting a PDGFRβ-STAT5-IGF1-SOCS2 signaling cascade that, at least in part, may drive the cataractogenesis phenotype observed in these mice (see model in Fig. 6). This proposed mechanism is supported by a separate study which, instead of the *Fsp1* promoter, utilized a skeletal stem cell specific promoter to drive Cre recombinase expression, and thus *Pdgfrb*^+*/D849V*^ hyperactivity, in skeletal stem cells (Kwon et al., 2021). In these mice, Socs2 was shown to play a role in skeletal stem cell fate through the STAT5-IGF1 axis, specifically that *Socs2*/*Igf1* overexpression and increased STAT5 activation led to increased chondrogenesis rather than osteogenesis, highlighting a possible aberrant lineage differentiation mechanism for the *Fsp1-cre;Pdgfrb*^+*/D849V*^ mice (Kwon et al., 2021). In support of this postulate, cataracts have been observed following induction of chondrogenesis in lenses from human donors, which become cataractous through collagen fibril deposition and stain positive for several markers of chondrogenic differentiation (Boix-Lemonche et al., 2023). Future experiments will probe for and quantify markers of chondrogenesis in *Fsp1-cre;Pdgfrb*^+*/D849V*^ lenses across cataract progression.

Of note, in the human eye, Socs2 is normally restricted to vascular and lymphatic endothelial cells (van Zyl et al., 2022). Yet, we observed dramatic overexpression of *Socs2* in *Fsp1-cre;Pdgfrb*^+*/D849V*^ lenses. It is important to acknowledge that for lens dissection, care was taken to remove the ciliary body and corresponding vasculature off the lens capsule, but complete removal is technically challenging. As such, there is the possibility that gene expression signatures, and thus high *Socs2*, from unremoved vasculature and ciliary body cells could confound our transcriptomic analysis. That said, regression of the hyaloid vasculature in the mouse begins on postnatal day 4 and there is substantial loss of the hyaloid vessels by day 10–12, with complete regression on day 21 (Wang et al., 2019). Thus, even considering this possible technical limitation, the observed upregulation of *Socs2* expression in *Fsp1-cre; Pdgfrb*^+*/D849V*^ mice is likely due to changes within the lens and not due to remnants of the hyaloid vessels.

Beyond the observed gene expression changes in the *Fsp1-cre; Pdgfrb*^+*/D849V*^ lenses, we also investigated TGFβ due to it being found abundantly in the aqueous humor and having been well documented in playing a role in fibrotic cataract formation (Saika, 2006). In other organ systems such as the embryonic mouse heart, TGFβ-mediated SMAD activation upregulates PDGFRβ expression to facilitate the EMT necessary for normal cardiac development (Peng et al., 2017). Therefore, it is reasonable to hypothesize that PDGFRβ may have downstream effects on TGFβ activity. Although we did not observe a statistically significant difference in *Tgfb1* expression at 10–12 days old, we did observe a significant upregulation in its expression in the lenses of *Fsp1-cre; Pdgfrb*^+*/D849V*^ adult mice at 15-weeks, along with increased SMAD2 activation. TGFβ has long been implicated as a master regulator of EMT-induced cataract, as well as in PCO (Saika et al., 2002, 2004; Wormstone et al., 2002). TGFβ has also been shown to induce Wnt expression and subsequent increases in β-catenin signaling during cataract development (Chong et al., 2009). Moreover, ectopic Wnt/β-catenin signaling has been shown to induce cataract formation in mice (Antosova et al., 2013). As such, we also investigated changes in *Wnt* expression and β-catenin stabilization in lenses of 15-week old *Fsp1-cre;Pdgfrb*^+*/D849V*^ mice, and observed a significant upregulation of *Wnt5b* and increased β-catenin in experimental lenses, indicating a secondary pathway involved in TGFβ-induced fibrosis. Future experiments should investigate the link between increased PDGFRβ signaling and activation of canonical TGFβ-induced fibrosis during cataract formation (Fig. 6).

Taken together, the results of the current study indicate that PDGFRβ hyperactivity mediates lens fibrosis through LEC activation and ECM remodeling, which when forced in a transgenic animal, appears at an early age by *Lama2* and *Socs2* dysregulation and is further potentiated by increased activation of TGFβ-SMAD2, Wnt/β-catenin, and STAT5-IGF1 signaling in young adult mice. Interestingly, we observe obvious cataract formation beginning at 9-weeks of age. As such, there may be mechanisms which we have yet to identify that delay or protect against cataract formation in mice younger than 9-weeks of age. We also observe that the cataract formation continues to progress beyond 15 weeks of age in the *Fsp1-cre;Pdgfrb*^+*/D849V*^ mice suggesting that pro-cataract signaling continues throughout life in these animals. However, the exact mechanism by which these cataracts worsen over time is not addressed here. Future studies exploring PDGFRβ in cataract formation, including in progressing cataracts, are necessary to yield new insight into cataract pathology and could inform the development of future therapeutics. Specific inhibition of PDGFRβ signaling may be efficacious as blocking this signaling in the lens could alter downstream signaling (TGFβ-SMAD2, Wnt/β-catenin, and STAT5-IGF1) to limit cataract formation and/or severity. Lastly, the findings presented herein provide evidence that the *Fsp1-cre;Pdgfrb*^+*/D849V*^ mouse model may prove as a valuable experimental tool for studying cataract formation in mice.

## Figures and Tables

**Table 1:** Significant differentially expressed genes in *Fsp1-cre;Pdgfrb*^+*/D849V*^ versus age-matched control lenses and gene annotations (n = 12/group).

Gene	Log_2FC_	padj	Notes
*Rps26-ps1*	−2.5926	1.36E-05	Ribosomal protein S26 pseudogene
*Lama2*	−1.05853	0.000352	Laminin subunit alpha 2, ECM protein regulating fate commitment of mesenchymal stem cells via hedgehog signaling
*Gm8186*	−2.50244	0.000352	Small nuclear ribonucleoprotein polypeptide G pseudogene
*Gm7536*	−1.03048	0.000511	Ribosomal protein L27A pseudogene
*1500015A07Rik*	2.025674	0.003086	RIKEN cDNA 1500015A07 gene, ncRNA
*Grik1*	1.553483	0.02339	Glutamate ionotropic receptor kainite type subunit 1, kainite family of glutamate receptors
*Gm10123*	2.143465	0.033781	Predicted pseudogene 10123
*Socs2*	2.503693	0.044093	Suppressor of cytokine signaling 2, negative regulator of cytokine receptor signaling, interacts with major signaling complexes to block transduction
*Slc7a2*	−0.52919	0.044093	Solute carrier family 7, involved in L-amino acid transmembrane transport and macrophage activation

## Data Availability

RNA sequencing raw data is available through the Gene Expression Omnibus (accession no. GSE302408).
